# Optimising adolescent wellbeing in a digital age

**DOI:** 10.1136/bmj-2021-068279

**Published:** 2023-03-20

**Authors:** Louise Holly, Brian Li Han Wong, Robin van Kessel, Isang Awah, Anurag Agrawal, Njide Ndili

**Affiliations:** 1 Governing Health Futures 2030 Commission, Global Health Centre, Graduate Institute of International and Development Studies, Geneva, Switzerland; 2 International Digital Health and AI Research Collaborative, Geneva; 3 Department of International Health, Care and Public Health Research Institute, Maastricht University, Maastricht, Netherlands; 4 LSE Health, Department of Health Policy, London School of Economics and Political Science, London, UK; 5 Global Parenting Initiative, Department of Social Policy and Intervention, University of Oxford, Oxford, UK; 6 Trivedi School of Biosciences, Ashoka University, Sonipat, Haryana, India; 7 PharmAccess Foundation, Lagos, Nigeria

## Abstract

Empowering adolescents and strengthening governance of digital media are among the urgent actions required to tackle the digital determinants of adolescent wellbeing, argue **Louise Holly** and **colleagues**

Today’s adolescents are transitioning from childhood to adulthood in an era of rapid digitalisation. Digital media—such as online communication platforms and applications accessed through mobile phones, tablets, and computers—are an important window into the world for adolescents. While levels of digital access and experiences of the digital world vary widely, adolescents are typically among the highest users of digital media.^1^ Roughly one in three internet users globally are under the age of 18.^2^ Young people often rely on smartphones to go online, but one third of school age children also have a fixed internet connection at home—rising to 88% in high income countries.^3^ Younger people also tend to spend more time online than older generations.^4^

As major users of digital media, adolescents are particularly exposed to their potential benefits and harms.^5^ More evidence is needed on the impact of digital technology use on adolescent development. Nevertheless, experiences during adolescence are widely accepted as having both positive and negative consequences for health and wellbeing throughout the lifecourse.^6^ Interactions with digital media during this critical period of physical and emotional development form part of the foundation for their future wellbeing as much as their interactions in the physical world.

To optimise adolescent wellbeing in this digital age, adolescents must be equipped and empowered to use digital media in ways that foster their immediate and future wellbeing. However, the burden of managing the risks of poorly regulated digital environments cannot be placed entirely on adolescents and their caregivers. Governance mechanisms and services for adolescents—including health services—must also be strengthened to provide the necessary support and protection to young users.^7^

## Digital determinants of adolescent wellbeing

Digital transformations—that is, the integration of digital technologies and data analytics into all areas of life—intersect with the social, political, commercial, and environmental determinants of health and wellbeing.^8^ ^9^ ^10^ ^11^ Recognising the multiple ways in which our health and wellbeing can be directly and indirectly influenced by digital transformations, a Lancet and Financial Times Commission has called for greater recognition of the digital determinants and action to tackle them.^12^

**Fig 1:**
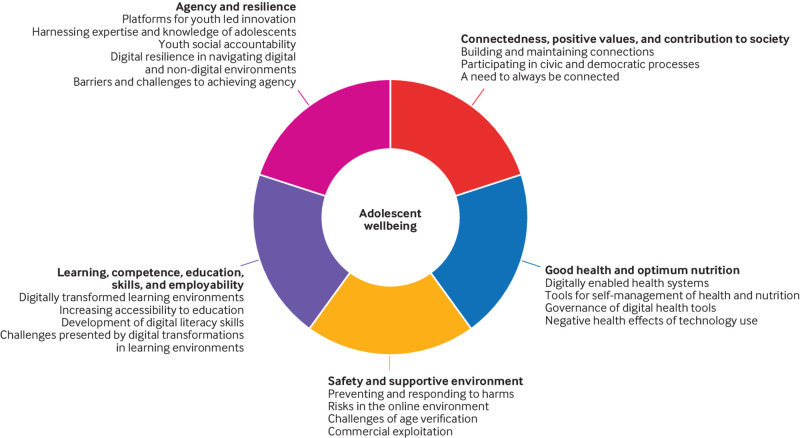
How digital transformations intersect with five domains of adolescent wellbeing

A framework specifically developed for adolescent wellbeing encompasses five domains: connectedness, positive values, and contribution to society; good health and optimum nutrition; safety and a supportive environment; learning, competence, education, skills, and employability; and agency and resilience.^13^ Digital determinants can positively and negatively affect adolescent wellbeing across each of these domains (fig 1).^5^

Digital media can improve adolescents’ wellbeing in multiple ways. For example, digitally enabled and data driven health systems can help to reduce health inequities and improve quality of care.^14^ Digital healthcare offers opportunities for health professionals to reach adolescents in remote and underserved communities and for adolescents to self-manage and monitor their physical and mental health. Online access to health related information is a major benefit identified by young people.^12^

Digital media platforms can support connectedness by allowing adolescents to sustain relationships with friends and family, make new social connections, pursue their interests, and find support networks. Adolescents use digital media to build their sense of identity, purpose, and fulfilment, and to contribute to their communities. Both formal and informal learning can be enhanced through digital media as adolescents gain access to unprecedented amounts of information and tools to build their knowledge and skills.

However, these potential benefits for adolescents are yet to be fully realised. Many adolescents, particularly those living in rural communities in low and middle income countries, do not have reliable or affordable internet access.^1^ Furthermore, the views, needs, and rights of adolescents are often overlooked in the design of digital tools and services.^12^ This persistent oversight by technology companies and governments has allowed digital media to evolve without adequate consideration of adolescents as users. A newspaper investigation found that TikTok and Instagram are exposing adolescents to inaccurate health information and harmful content.^15^ Testifying at the US Congress in December 2021, the head of Instagram admitted that this social media platform, which is popular among adolescents, “wasn’t designed for them.”^16^

A range of risks can arise through adolescent engagement with digital media (box 1). Some design features of digital media can encourage risk taking and promote unhealthy behaviours. “Hyperconnectivity” through digital media may displace in-person relationships and healthy behaviours such as physical exercise.^17^ Young people are especially concerned about exposure to harmful content, the reliability of information online, and data privacy.^12^ Better evidence is needed on how these risks could result in physical, mental, or emotional harm in adolescents, especially in low and middle income countries. Nevertheless, given the potential severity of harm to adolescents’ health and wellbeing, pre-emptive action is needed.

Box 1 **Categories of risks that adolescents are exposed to when using digital media^18^**

*Content risks*—Adolescent engages with, or is exposed to, potentially harmful content, including violent or pornographic material; discriminatory, hateful, or extremist information; content that perpetuates harmful body image norms; misinformation or disinformation; or age inappropriate marketing
*Contact risks*—Adolescent experiences or is targeted by potentially harmful adult contact resulting in harassment; unwanted surveillance; sexual abuse; or ideological persuasion, manipulation, or radicalisation
*Conduct risks*—Adolescent witnesses, participates in, or is a victim of potentially harmful peer contact, resulting in bullying, hostile communication or peer activity (eg, trolling), sexual harassment, non-consensual messaging, or harmful user communities (eg, self-harming or dangerous online challenges)
*Contract risks—*Adolescent is party to, or exploited by, potentially harmful contracts such as identity theft, fraud, online scams, trafficking for sexual exploitation, gambling, micro-targeting of content, and marketing practices that shape behaviour or purchases
*Other cross cutting risks*—This category includes privacy violations (interpersonal, institutional, commercial), negative physical and mental health impacts of excessive digital media use, and inequalities and discrimination resulting from levels of access to digital media, algorithmic bias, or predictive analytics

Adapted from The 4Cs: Classifying Online Risk to Children^18^

## Need for coordinated action at different levels

Adolescents say that they want to be protected online but do not want their access to digital media to be restricted. Results from consultations with 709 children and young people aged 9-22 years in 27 countries highlight how they want spaces to be imaginative, to experiment, and to exercise their agency, but they also want digital platforms to stop commercially exploiting them, collecting their data, and exposing them to misinformation and harmful content.^19^

How adolescents navigate the opportunities and risks associated with digital media depends on a wide range of individual level factors, including their digital literacy and their wider social, economic, and political environment.^5^ Tackling the digital determinants of adolescent wellbeing therefore requires a whole-of-society approach and action at multiple levels. Adolescents’ needs and demands can be met through a coordinated, multisectoral response to the digital determinants of adolescent wellbeing that includes strengthening governance of digital media; increasing adolescents’ agency, literacy, and skills; and building the health sector’s capacity to respond to the evolving role of digital media for adolescent wellbeing.

## Strengthening regulation to protect adolescents online

Governance of digital media has fallen behind the pace of technological innovation and the evidence base on how digitalisation can support or harm adolescent wellbeing. Action to strengthen governance, such as legislation at national level or self-regulation by technology companies, is often reactive and taken only after a serious incident, such as the preventable death of an adolescent and ensuing media coverage.^20^ ^21^ Box 2 summarises some examples of approaches introduced by prominent digital media companies to protect children and adolescents.

Box 2 **Common approaches to protecting children and adolescents online**

*Age assurance* **—**An umbrella term for approaches that are designed to ensure users of a product or service are a certain age. While European Union legislation, for example, does not require users of digital products and online platforms to be a certain age, they may require a distinction to be made between children and adults, given the vulnerability of children in view of their age and development.^22^ Many popular social media platforms use age assurance tools to restrict access to children under 13 (following the definition of a child used in the US Child Online Protection Privacy Act)
*Data consent pop-ups and cookies* **—**A system used by most websites for acquiring user consent for data tracking in online advertising. With the introduction of the General Data Protection Regulation in the European Union, the use of these data pop-ups has increased to ensure continuation of service provision. However, in a 2022 cornerstone ruling, these pop-ups have been found to breach the General Data Protection Regulation as they do not sufficiently inform the subject how their data are handled and processed^23^
*Artificial intelligence (AI) monitoring* **—**Currently most widely known for its application in combating covid-19 misinformation, AI is already widely used to detect potentially harmful content (eg, explicit, triggering, violent, abusive, and radicalising content) on social platforms such as Facebook, YouTube, Instagram, and LinkedIn^24^
*Discouraging excessive use* **—**Some digital products have included provisions to discourage excessive or prolonged use. For instance, YouTube disabled autoplay for younger users; Google turned off targeted advertising and tracking for minors; and Facebook, Instagram, and TikTok made similar concessions.^25^ Some video games and platforms hosting games, such as Tencent, have introduced systems that slow down or halt progression after a certain amount of time of playing consecutively (colloquially named “fatigue systems”) to combat excessive use^26^

Many of the protective measures being introduced by governments and technology companies place a high burden of responsibility on adolescents and their caregivers to understand and then correctly apply the recommended controls. For example, the detailed terms, conditions, and cookie preference pop-ups that users are now invited to review each time they go online assume unrealistic levels of digital literacy. Other approaches, such as “time spent online” warnings in video games, may be easily ignored if the risks of prolonged use of digital media are unknown, which may be common given recent estimates of digital literacy levels.^27^

As well as trying to shift user behaviour, policy makers are increasingly recognising that digital media also need to change to align better with the best interests and rights of children and adolescents.^21^ Many countries have introduced legislation and other regulatory approaches to make digital environments more suitable for young people. While some countries censor the activities of digital media companies or limit young people’s access, others, including Australia, Germany, Singapore, the United Kingdom, and the United States, are pursuing approaches that champion child centred design of digital technologies and seek to protect children and adolescents within the digital world rather than restricting them from it.^28^ ^29^ ^30^ ^31^ ^32^ ^33^

## Empowering adolescents to reap the benefits of digitalisation

All countries can do more to strengthen governance of digital media and ensure they offer better experiences for young users. A holistic interpretation of adolescent wellbeing must be central to all approaches. Protective and regulatory measures alone are insufficient for maximising adolescent wellbeing; their need for connectedness, learning, and agency must also be considered. Particularly during the covid-19 pandemic, exclusion from the online world has been shown to have a detrimental impact on adolescents’ ability to continue learning, stay connected to friends and family, express themselves, and be active citizens.^3^ Consequently, to improve wellbeing governments must make digital environments more adolescent friendly while simultaneously reducing digital divides.

Not all parts of the internet can be made safe, and young people will often find ways around even the tightest guardrails.^34^ Adolescents will truly be safe online only when risks are minimised and they can identify and deal with any remaining risks themselves. To confidently enjoy the advantages offered by digital transformations and know what to do if they encounter harmful content or practices, all adolescents must have a comprehensive digital education.

Digital skills are essential for adolescents to learn, access services, and prepare for work. A recent systematic review of the outcomes of gaining digital skills during adolescence found a positive association between early acquisition of digital skills and online opportunities, information benefits, and orientation to technology related career paths.^35^ A range of initiatives to increase young people’s digital literacy and skills are being implemented by governments and non-governmental organisations, but more systematic, population level education and civic programmes are needed to reduce inequities in digital literacy and opportunities.^36^ Further evidence is also needed on the impact of investment in building adolescents’ digital literacy and skills to understand which approaches are most effective.

Adolescents have a right to participate in decisions that affect their lives, which include decisions pertaining to the digital world.^37^ Governments can enhance adolescents’ sense of agency by empowering and enfranchising them to participate in the design, use, and governance of digital media and technologies. Besides having a huge stake in the outcomes of governance decisions, adolescents’ unique experiences and perspectives are critical for building a digital world fit for its youngest inhabitants.^38^

## Health sector’s role in optimising adolescents’ digital media use

Digitalisation and digital media are now interwoven into all parts of our lives, and tackling their impact must be a priority for all sectors. Given the potential for health and wellbeing to be both enhanced and undermined by digital media, the health sector must work with other sectors to ensure that they are designed and used in ways that align with health goals.

Health professionals have a vital role in signposting adolescents and their caregivers to verified sources of online health information and support in the event of risky or harmful digital media use. To enable them to offer the best possible care and advice, health professionals working with adolescents should receive regular training to keep pace with fast changing digital innovations, the different ways that adolescents use digital media, and the positive and negative implications of digital media use for health and wellbeing.

As a result of their experiences working directly with adolescents, health professionals can effectively advocate for stronger, more adolescent centred governance of digital media, and for greater investment in population level digital education programmes. Health professionals are uniquely positioned to monitor and gather evidence on the impact of adolescent digital media use and to push for evidence based policies and programmes.

## Conclusion

Tackling the digital determinants of adolescent wellbeing requires proactive multisectoral action. Removing barriers to digital access for adolescents, equipping them with digital literacy and skills, and giving them opportunities to shape their digital environments will enable them to enjoy the full benefits of digital transformations and enhance their wellbeing. Governments and technology companies must fulfil their obligations to putting adolescent wellbeing at the heart of digital design and governance.

Improving wellbeing has immediate and long term benefits for adolescents, and these benefits will be passed on to future generations.^39^ As the role of digital media in young peoples’ lives grows, a holistic and rights respecting approach to promoting their wellbeing both online and offline becomes increasingly necessary to enhance the lives of adolescents and wider society.

**Key messages**

- Digital media play an integral part in many adolescents’ transition from childhood to adulthood
- Digital transformations—the integration of digital technologies and data analytics into all areas of life—can directly and indirectly affect adolescent wellbeing in both positive and negative ways
- Tackling the digital determinants of adolescent wellbeing requires coordinated action to empower adolescents, strengthen governance of digital media, and build the capacity of the health sector to respond to the evolving role of digital media for adolescent wellbeing
- Health professionals can have an important role in supporting adolescents to use digital media in ways that promote their wellbeing and in encouraging governments to put adolescent wellbeing at the forefront of digital governance

## Notes

Contributors and sources:The authors have expertise in global health policy and governance and have conducted analysis and research on the impact of digital transformations in health, particularly for children, adolescents, and youth. AA is co-chair of the *Lancet* and *Financial Times* Commission on governing health futures 2030 and NN is a commissioner. LH and BLHW are part of the commission’s secretariat. LH led on the conceptualisation and writing of the original draft of the manuscript. LH, RVK, and BLHW made equal contributions to the formal analysis. RVK and BLHW made equal contributions to the conceptualisation, review, and editing of the manuscript. IA reviewed, edited, and contributed to the manuscript. NN and AA reviewed and contributed to the manuscript.

Provenance and peer review: Commissioned; externally peer reviewed.

This article is part of collection proposed by the Partnership for Maternal Newborn and Child Health. Open access fees were funded by the Bill and Melinda Gates Foundation. The BMJ commissioned, peer reviewed, edited, and made the decision to publish these articles. Emma Veitch was the lead editor for The BMJ.
